# Two Cases of Puerperal Convulsions

**Published:** 1858-06

**Authors:** J. Williamson

**Affiliations:** Ottumwa, Iowa


### TWO CASES OF PUERPERAL CONVULSIONS.

BY J. WILLIAMSON, M. D., OTTUMWA, IOWA.

On the morning of the 22d of April, 1857, I was sent for to see a patient, Mrs. C., in consultation with Dr. A. D. Wood. Upon arriving I found he had a case of puerperal convulsions, the first fit having occurred about an hour previous to my arrival. The patient was a primipara, of about 20 years of age, nervo-sanguine temperament, rather plethoric, and of strong muscular fibre. The patient, as I learned from the Dr., had been in labor about six hours, the os uteri was slow to dilate, presentation natural, and nothing out of the usual course of labor was anticipated, except, perhaps, it might be somewhat protracted, the liquor amnii having passed off in the early part of the labor.

The patient had had two convulsions previous to my arrival, upon the subsidence of the first she had been bled XL. ounces, a second having come on Dr. W. was trying the effect of chloroform. The patient was in deep coma from which she could not be aroused in the interval of the convulsions, pulse 130, deep, stertorous breathing, frothing at the mouth, face and neck greatly swollen. Labor not having progressed so far as to prevent turning of the child, it was suggested that as now the mouth of the womb would offer but little resistance to the introduction of the hand, we turn and deliver. At the request of Dr. Wood I proceeded to make the attempt, not, however, without some misgivings as to the success of the effort, in view of the waters having passed off, as before stated, in the early part of labor. Fortunately, however, there was no difficulty in pushing up the head, which was now engaging in the superior strait, and bringing down the feet and thus completing delivery in about twenty minutes. The child was dead. She lost but little blood in delivery. The convulsions continued to recur every 30 or 40 minutes; pulse rose to 140. I suggested the use of the Veratrum Viride in doses sufficient to bring down the pulse in the shortest time compatible with safety. Gave Norwood's tincture, eight drops, repeated doses of five drops each, every 40 or 60 minutes, until pulse was reduced to 90 which required six doses, after which it was used less freely. Meantime we gave morphine in half grain doses every hour. Chloroform was also used until satisfied that it would not obviate the convulsions or even mitigate their severity. The remedies then upon which we relied were V. V. and morphine. From 5 o'clock, A. M., which was her first, until 2 o'clock, P. M., she had ten convulsions as severe as can well be conceived; after which time they did not return. By this time our patient was the most deplorable looking object I ever beheld. Tongue enormously swollen and protruded, rendering it difficult to administer the medicine; face and neck bloated, eyes rolled back, and her whole physiognomy presenting a glassy and cadaverous appearance. But I need not dwell upon appearances when the bare mention of the disease to every intelligent physician, suggests a train of symptoms to his mind the most terrible and hopeless of any that befall the puerperal patient.

The treatment for the night was V. V. five drops, every 4 or 6 hours, with such attention to her person as was admissible without occasioning too much disturbance and agitation.

Next morning I found our patient better and able to signify her desires, pulse 40, face and neck less swollen, complained of nothing but soreness. From this time she convalesced without embarrassment and with little other treatment than is usually bestowed upon the parturient patient.

Case 2. On the evening of the 15th of Feb. I was requested to visit Mrs. G., who was then in labor with her first child. Patient 18 years of age, of same temperament and frame as Mrs. C. whose case is given above. Labor progressed slowly. After two and a half or three hours, an examination revealed a vertex presentation, womb partially dilated, parts moist and disposed to yield. I sat down to await dame nature's fullness of time, little doubting but she would show herself entirely competent to the management of the case. As the pains became stronger and the head engaged in the superior strait, the patient became restless and uneasy. From two to three hours wore away with comparatively little advance of the head. At this stage of labor with no discouragement in view, and with fair prospects of delivery in good time, during a pain I noticed a tremor of the muscles of the limbs, a moment more and my patient was in a convulsion. Perhaps for the first time in my professional life, I found myself without a lancet. I requested the assistance of my partner, Dr. S. B. Thrall, with directions to bring my forceps. In less than thirty minutes from the time of the first convulsion the doctor was present, and while in the act of bleeding her, she had a second one. We bled her to the amount of XL ounces. In the interval of the convulsions she was totally unconscious and tossing herself about, requiring the assistance of two or three to keep her on the bed. So violent indeed were her involuntary movements, that three strong assistants were unable to maintain the requisite quiet for the successful application of the forceps. After spending some minutes in ineffectual efforts to restrain her by physical force, we resorted to the use of chloroform. A few inhalations rendered her entirely passive in our hands. A more careful examination now disclosed that the child was presenting in the fifth position of Ramsbotham; face to the right groin; occiput to the left sacro-iliac synchondrosis; right ear to the left groin, and left ear to right sacro-iliac synchondrosis. Maintaining perfect quiet by the use of the chloroform, I proceeded to apply the forceps and deliver, which was readily done. The child was dead. It is worthy of note that the mother had felt no motions of the child for three weeks previous, but we saw no indications of its having been dead previously to the commencement of labor. She was now put to bed and given a grain and a half of acetate of morphine. I should estimate the loss of blood in delivery as equal in amount to that taken from the arm. Pulse was now 120 and easily compressed. After delivery she had three convulsions at intervals of forty or fifty minutes, each becoming less severe than the preceding. The first eight hours after the completion of labor no other treatment was directed than half grain doses of morphine every two hours, after which gave Veratrum Viride, five drops every three hours until pulse should be reduced to natural standard.

Since treating the case of Mrs. C., we had seen several cases of alarming prostration from what we thought the judicious use of the V. V., and we feared the result of such powerful sedation as the abstraction of XLV ounces of blood followed immediately by the use of this article. Twelve hours sufficed to bring down the pulse and we persevered in its use without intermission until the fourth day. This, with some attention to the bowels and an occasional dose of morphine, was the sum of the treatment until the eighth day, when a severe and protracted ephemeral chill occurred which created some alarm to the friends, but which readily yielded to the usual remedies. She is at this time, the 17th day, since her delivery, able to be up, and is fast regaining her accustomed strength.

In view of the number of cases of Puerperal Convulsion that have occurred in this community within the last few years, the inquiry arises, can there be any local or endemic cause for it? The last four years have brought four cases under my own observation, and I have learned upon conferring with the physicians of this place, that there have been in the time stated, no less than *twelve* cases in the town and vicinity. Eight of these were first labors. In ten the convulsions came up prior to delivery; in the eleventh the attack followed in about half an hour after patient had been put to bed; in the twelfth, ten hours had elapsed before any symptoms appeared. Nine were delivered without manual assistance; one died undelivered. Four of the children were still born; one died in an hour or two after birth. Two of the cases were followed by mania, one on the second day, (which died on the sixth,) the other on the fourteenth, recovered. In the winter of '48–9, Prof. Delemater remarked to his class that, in a practice of thirty-five years he had not met with a single case. I have met other physicians of fifteen and twenty years practice, who have met with no cases. It is hardly possible that this has been owing to a closer vigilance in detecting the approach and warding off the attack. In some, at least, of the cases above alluded to, there were none of the ordinary premonitions.

The extraordinary frequency of convulsive diseases among children, has been a subject of remark among physicians of this place.

I would here in a word bear my testimony to the incomparable virtues of the Veratrum Viride in all cases where the indication is to abate the frequency and force of the pulse. There is no other remedy of which we can say in reference to the heart's action, thus far shalt thou go and no farther. Witness the effect in the case of Mrs. C., where in eighteen hours, the pulse fell one hundred beats in the minute. Within a year past, I have had opportunity to witness its good effects in a considerable variety of diseases, in which the leading indication was to bring down the pulse. In small pox, where the amount of the eruption, which, to a great degree, is the measure of the impending danger, is in a direct ratio to the violence of the primary fever, I consider it the remedy above any other. In my use of it, Scarlatina has formed an exception to the general statement above given. In thirty or forty cases in which I used it, as I thought freely, I saw no diminution in the frequency of the pulse, still I am not prepared to say that others would not have succeeded better. But granting that it will assert its peculiar powers here, I am led to believe that, in this, as in other diseases, arising from a specific cause, its good effects are limited chiefly to the avoidance of inflammatory complications, the elimination of the *materies morbi* being the end to which our remedies must be directed.

